# Clinical characteristics and pathogen spectra of parasitic infections in a tertiary hospital of Shanghai: A 13-year retrospective study

**DOI:** 10.3389/fpubh.2022.993377

**Published:** 2022-09-28

**Authors:** Jinming Zhang, Jing Xu, Weiliang Tang, Ruidong Mo, Dabao Shang, Jie Lu, Ziqiang Li, Xiaolin Wang, Dongmei Shi, Qing Xie, Xiaogang Xiang

**Affiliations:** ^1^Department of Infectious Diseases, Ruijin Hospital, Shanghai Jiao Tong University School of Medicine, Shanghai, China; ^2^Translational Laboratory of Liver Diseases, Department of Infectious Diseases, Ruijin Hospital, Shanghai Jiao Tong University School of Medicine, Shanghai, China; ^3^Department of Infectious Diseases, Lixin People's Hospital, Bozhou, China

**Keywords:** parasitic infection, food-born parasite, parasitic spectrum, *Clonorchis sinensis*, parasitic diagnosis

## Abstract

**Background:**

This study performed a follow-up investigation of parasitic infections and the evolution of the infection spectra in Shanghai and its surrounding areas in Eastern China. The current study was conducted in the Shanghai Ruijin Hospital, a tertiary hospital affiliated with Shanghai Jiao Tong University School of Medicine.

**Methods:**

This retrospective investigation reviewed a total of 412 parasitic infections in patients admitted to the Department of Infectious Diseases, Ruijin Hospital from January 1, 2010 to July 31, 2022. Detailed information for these patients was retrieved from the Electronic Medical Record System. Analysis was performed using GraphPad Prism 5.0 and SPSS Statistics 26.

**Results:**

Overall, 17 species of parasites were detected from the 412 admissions. Over the 13 years, the number of patients peaked in 2021 and food-born parasites (FBPs) were the primary species. During the most recent 5 years, *Clonorchis sinensis*, replacing *Paragonimus westermani*, has become the primary parasite detected among the patients, consistent with the observation that eating uncooked fish has turned into the most common route of transmission. *Paragonimus westermani* infections declined with age, but *Cysticercus* increased with age. The periods from the onset of symptoms to definite diagnosis for some patients infected with *Sparganum mansoni, Paragonimus westermani*, and *Cysticercus* were more than 6 months. Interestingly, eosinophilia was only detected in 51.83% of parasite-infected patients. In addition, superinfections of parasites were common in our study.

**Conclusion:**

Our study demonstrates the transitional change in the prevalence of parasitic infection over the latest 13 years in a single center in Eastern China. The incidence of parasitic infections peaked in 2021, and the dominant parasitic species switched from a soil origin to foodborne. The direction for the diagnosis and prevention of parasitic infection among different age groups should alter according to age. It is difficult to diagnose parasitic infections and superinfections that occur in some patients. Thus, more sensitive and efficient detection methods should be developed. In addition, although eosinophilia and elevated IgE are still reliable indicators for initiating screening of parasitic infection, the development of novel parasitic diagnostic kits is still in urgent need for occult infection.

## Introduction

In the early 1960s, there were many parasitic infections that seriously impacted the health of people worldwide, especially in China (1, 2). Parasitic infection was problematic and a huge burden on economic development (3, 4). Despite the exceptional improvements that have been accomplished in fighting parasites over the last 60 years in China, parasitic infections still account for a large percentage of the burden (1). Soil-transmitted parasites and food-born parasites (FBPs) are the two dominant categories of parasites. Because FBPs frequently contaminate animal meat or aquatic products, the main routes of parasite transmission are eating raw or uncooked food that is contaminated with parasitic eggs or larvae (5). Importantly, soil-transmitted parasites (also named soil-transmitted nematodes) do not require an intermediate host, and their eggs and/or larvae that contaminate soil are able to transmit these parasites directly to humans (6). Recent alterations in the routes of transmission reflect the current situation of economic development of a country to a certain extent, since there have been changes in the species spectrum of parasites over recent years following rapid advances in both the economy and sanitary conditions within China.

Recently there have been fewer epidemiological studies that have focused on parasitic infections and/or the transition of the forms of parasitic infection spectra in Eastern China (7), due to the substantially declining incidence of parasitic infection in developed regions. However, there are still some parasite-infected patients with complications referred from the local hospitals/clinics to our hospital, which is a tertiary teaching hospital with specialized experience in infectious diseases. These parasite-infected patients cannot be managed properly in their local hospitals. Thus, it is necessary to investigate the recent epidemiological features and transition of parasitic infections in the representative areas, especially in Shanghai. Detailing the transition of the parasitic spectra could provide valuable information for parasite infection diagnosis and prevention, such as the dominant parasites at present, the main routes of transmission, and the potential biomarkers for the diagnosis of parasitic diseases. The present study retrospectively investigated the prevalence of parasitic infections in a tertiary hospital in Shanghai from 2010 to 2022.

## Materials and methods

### Study design and subjects

**Figure 1:**
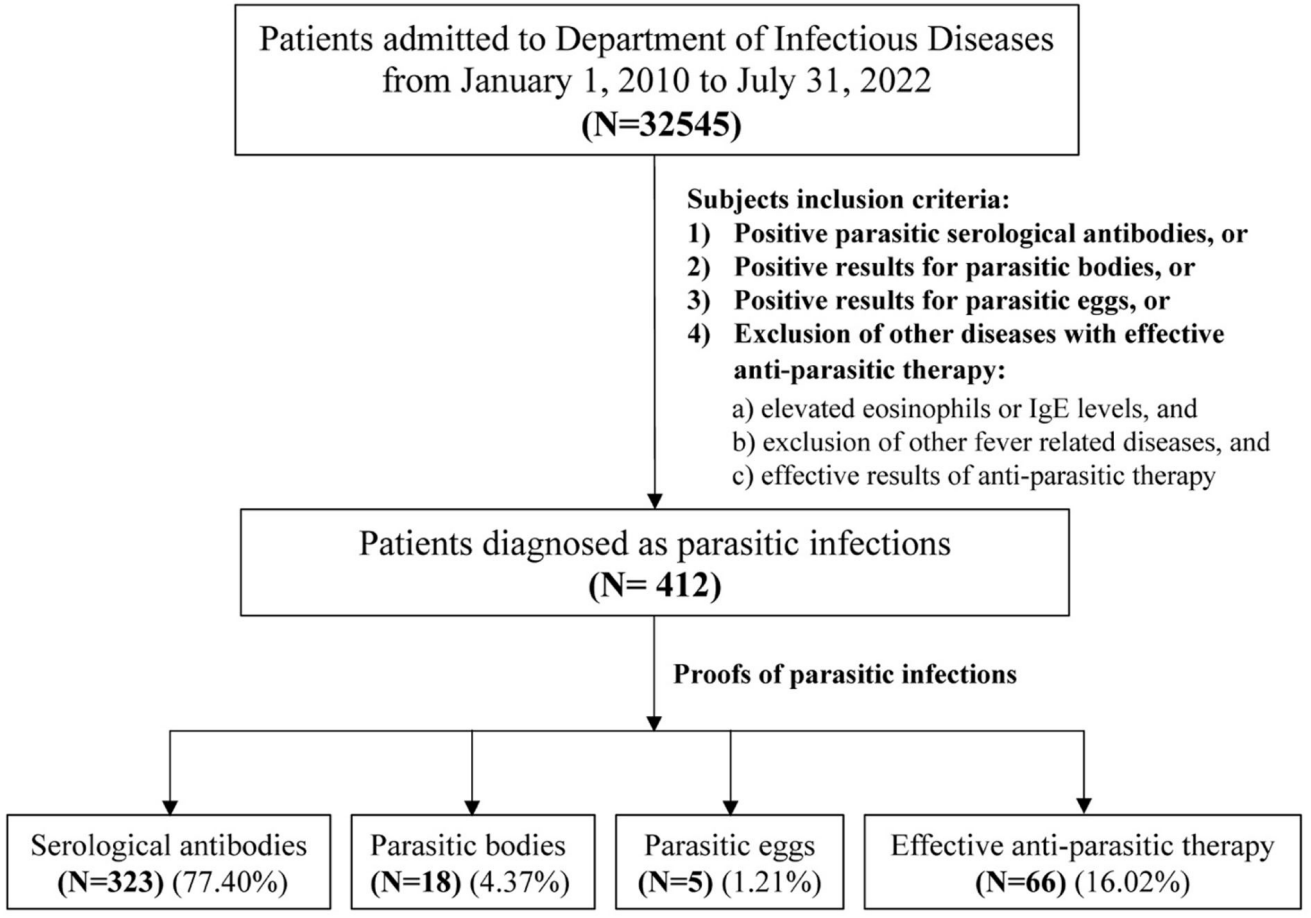
Flowchart of the subjects' inclusion criteria. Among total of 32545 patients admitted to the Department of Infectious Diseases of Ruijin hospital from January 1, 2010 to July 31, 2022, there were only 412 patients were parasitic infections. A small number of patients with uncertain parasitic infection (16.02%) were diagnosed by the exclusion of other diseases with effective anti-parasitic therapy, and all the following three criteria should be met in this group: (1) elevated eosinophils or IgE levels; (2) exclusion of other fever-related diseases; and (3) effective results of anti-parasitic therapy.

This single-center retrospective study was performed in the *Department of Infectious Diseases, Ruijin Hospital, Shanghai Jiao Tong University School of Medicine* in China from January 1, 2010 to July 31, 2022. Four hundred and twelve patients who were diagnosed with parasitic diseases, consisting of 166 female and 246 male patients, were identified (Figure 1). Parasitic infection was diagnosed with at least one of the following manifestations: (1) positive parasitic serological antibodies; or (2) positive identification of parasitic bodies; or (3) positive identification of parasitic eggs; or (4) exclusion of other diseases in the context of effective anti-parasitic therapy. Serological testing was undertaken by the laboratory from *The National Institute of Parasitic Disease, Chinese Center for Disease Control and Prevention* (CDC), using diagnostic kits developed by themselves based on IgG serology, except for *Toxoplasma* where both IgG and IgM serologies were used. Almost no laboratories within hospitals in China have such tests. Most of the patients were diagnosed using definitive laboratory results, while a small number of patients with uncertain parasitic infection evidence (16.79%) were diagnosed via a differential diagnosis following the exclusion of other diseases and effective anti-parasite therapy (diagnostic treatment). In this category, all the following three criteria must be met: (1) elevated eosinophils or IgE; and (2) exclusion of other fever-related diseases; and (3) effective results of anti-parasite therapy. The current study was carried out in accordance with *The Code of Ethics of the World Medical Association* (Declaration of Helsinki). Informed consent was obtained for experimentation with human subjects.

### Data collection and analysis

All clinical and diagnostic data of patients with parasitic infection from January 1, 2010 to July 31, 2022 were retrieved from the Electronic Medical Record System, *Ruijin Hospital*. The sera of patients for the first visit with clinical manifestations, such as fever of unknown origin (FUO) or elevated eosinophils or IgE levels were sent for parasitic diagnosis. After anti-parasitic treatment, those who visited our hospital with recurrent clinical manifestations (fever or elevated eosinophils/IgE levels) were considered to be a repeat infection or inpatient. The laboratory results were collected at the time of the first visit. The onset time of parasitic infection and the routes of transmission were collected during the consultation of patients. Dates of diagnosis were defined as the time when positive laboratory results supporting parasitic infections were obtained or anti-parasitic treatments were determined to be effective. Categorical variables were described as frequency or percentages, and continuous variables as mean and standard deviation (SD) if they were normally distributed or median and interquartile range (IQR) if not. Categorical variables were compared, using the χ^2^-test. Means for continuous variables were compared, using the independent group *t*-test between two groups when the data were normally distributed; otherwise, the Mann–Whitney U test was used. Kruskal–Wallis was used for comparing two or more independent samples among non-normally distributed data. *P* < 0.05 was considered significant. Data and basic information for patients were imported to Excel 2010 (Microsoft, Redmond, WA, USA) and analyzed by GraphPad Prism 5.0 (GraphPad Software, San Diego, CA, USA) and SPSS Statistics 26 (IBM, Armonk, NY, USA).

## Results

### Epidemiological characteristics of parasitic diseases

**Figure 2:**
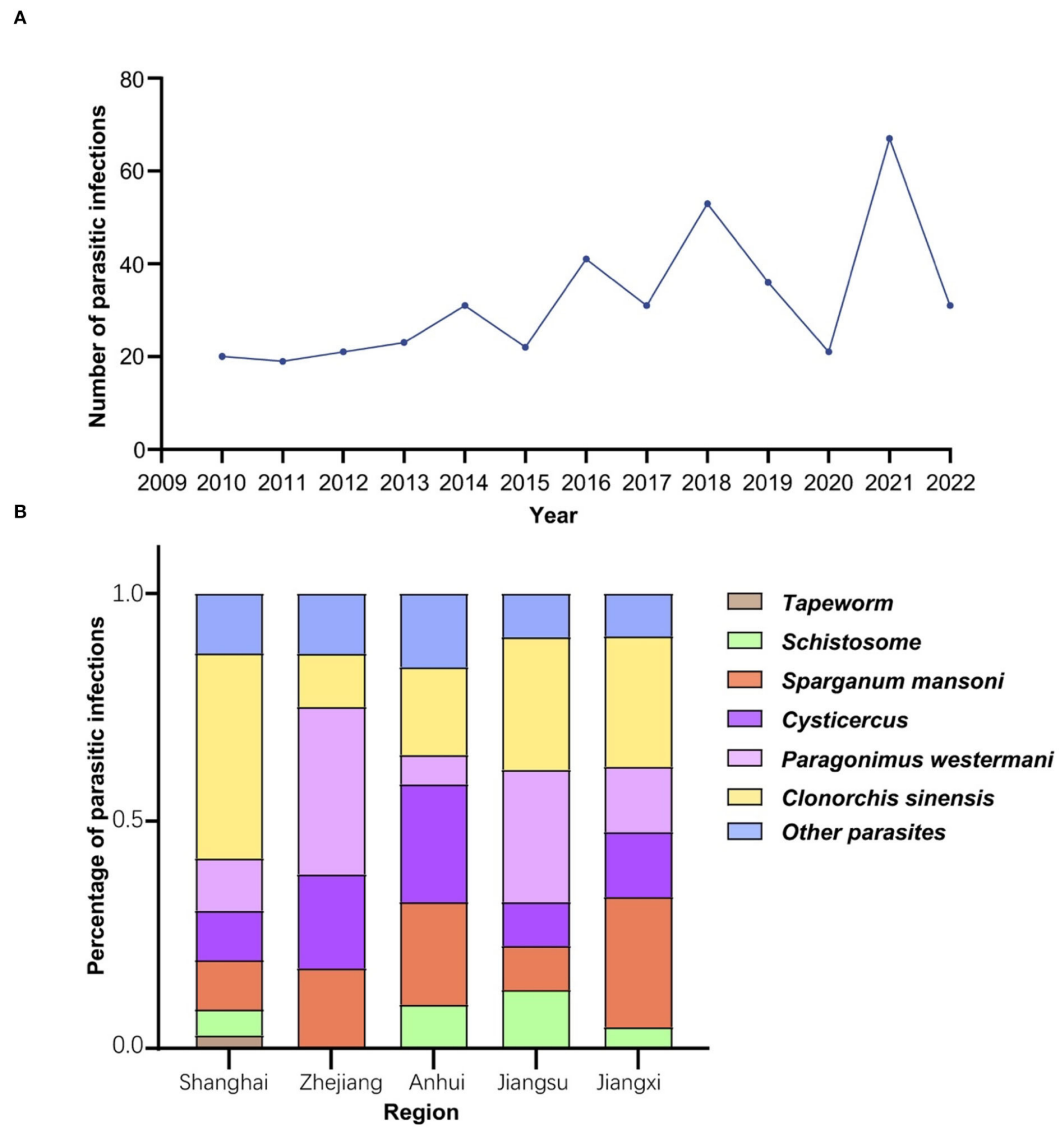
Epidemiological characteristics of parasitic diseases. **(A)** Parasitic infections in Ruijin hospital over the recent 13 years. The prevalence of parasitic infection showed an upward trend of waves with a peak in 2021. **(B)** The prevalence of the most common six parasites in five main regions of origin. Parasite-infected patients admitted to *Shanghai Ruijin Hospital* were mainly from Shanghai, Zhejiang, Anhui, Jiangsu, Jiangxi. *Clonorchis sinensis* was the most common parasite found in patients from Shanghai, while *Paragonimus westermani* was the most common one identified in patients from Zhejiang and Jiangsu.

Four hundred and twelve parasite-infected patients were identified for the current study. For the prevalence of parasitic infection over the years from 2010 to 2022, a gradual increase was demonstrated from 2010, which peaked in 2021, as follows 20, 19, 21, 23, 31, 22, 41, 31, 53, 36, 21, 63, and 31 cases each year from 2010 to 2022 (Figure 2A, Table 1). These patients identified were mainly from five regions: Shanghai (42.48%, 175/412), Zhejiang (16.26%, 67/412), Anhui (7.52%, 31/412), Jiangsu (7.52%, 31/412), and Jiangxi (5.10%, 21/412), accounting for 78.88% of the total infections (Table 2).

**Table 1:** Parasitic infections over recent 13 years in a tertiary hospital in Shanghai.

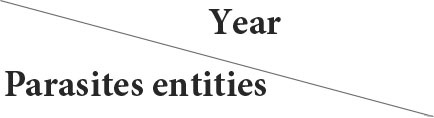	**2010**	**2011**	**2012**	**2013**	**2014**	**2015**	**2016**	**2017**	**2018**	**2019**	**2020**	**2021**	**2022**
Amoeba	1						1		1		1		
Hydatid					1	1		1	1			1	
Tapeworm	1	1			1	1	2	1	2	1	1	3	
Paragonimus westermani	3	7	6	7	2	1	15	11	7	8	3	6	
Toxoplasma gondii	2				3		1			1	2	2	1
Hookworm				1					1			3	1
Clonorchis sinensis	1	2	2	3	4	1	7	7	22	12	8	42	25
Borrelia burgdoferi				1		1							
Giardia lamblia									1				
Sparganum mansoni	4	4	3	2	3	4	10	4	11	7	2	2	3
Cysticercus	6	5	5	7	13	8	4	3	2	4	3	3	
Leishmania spp			1										
Plasmodium				1		1	1	3	1				
Blastocystis hominis	1		1					1	1				
Trichinella spiralis					2								
Schistosome			2	1	1	4			3	3	1	1	1
Tsutsugamushi	1		1		1								
Total	20	19	21	23	31	22	41	31	53	36	21	63	31

Seventeen species of parasites were found in our study from 2010 to 2022.

**Table 2:** The main origins of parasite-infected patients.

**Region**	**No. of patients (*n*)**	**Proportion of parasitic patients (%) (*n*/412)**
Shanghai	175	42.48
Zhejiang	67	16.26
Anhui	31	7.52
Jiangsu	31	7.52
Jiangxi	21	5.10

Parasite-infected patients admitted to the hospital were mainly from Shanghai, Zhejiang, Anhui, Jiangsu, and Jiangxi.

Patients from other regions numbered <10 from each region: i.e., nine from Guizhou, eight from Henan, eight from Heilongjiang, five from Jilin, five from Sichuan, four from Fujian, four from Hubei, four from Chongqing, three from Guangdong, three from Hunan, three from Liaoning, three from Shanxi, two from Guangxi, two from Inner Mongolia, two from Shandong, two from Xinjiang, one from Beijing, one from Hainan, one from Qinghai, one from Shanxi, one from Xizang, and one from Yunnan.

In addition, the prevalence of parasitic infections was significantly different in these five regions (χ^2^ = 68.46, df = 20, *P* < 0.0001) (Figure 2B). Infections with *Clonorchis sinensis* were the most common in Shanghai, while infections with *Paragonimus westermani* were the most common in Zhejiang and Jiangsu provinces.

### Demographic characteristics of parasite-infected patients and the main routes of transmission

**Figure 3:**
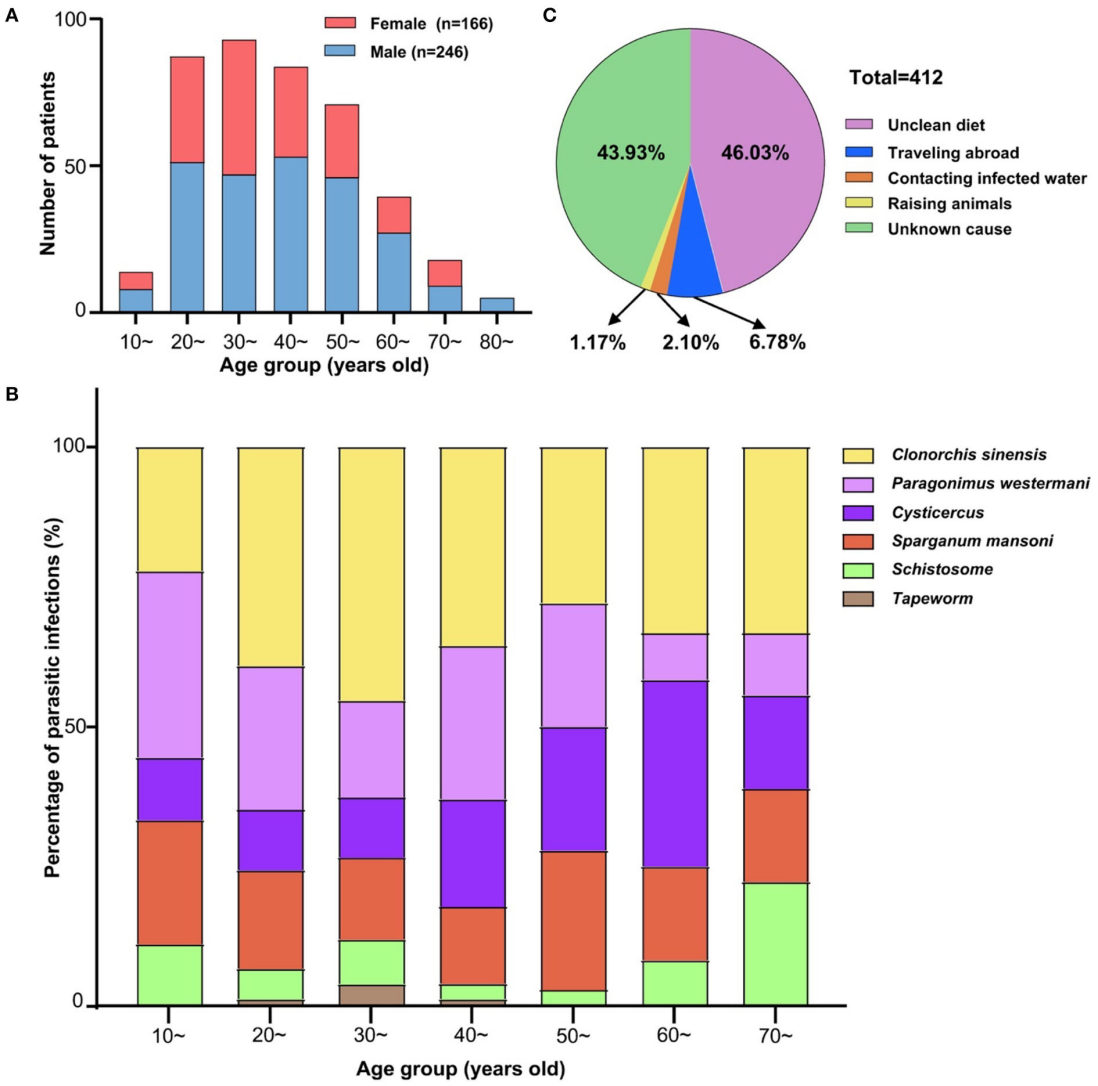
Demographic characteristics of parasite-infected patients and the main routes of transmission. **(A)** Gender and age distribution of parasite-infected patients. Four hundred and twelve parasite-infected patients were categorized into nine different age groups. Male vs. female ratio was 1.48:1 with the age of 95% of patients ranging from 21 to 70. **(B)** Parasitic species with top six infections in different age groups. The prevalence of the top six parasitic infections showed a significant difference among different age groups (χ^2^ = 64.1, df = 30, *P* < 0.001). The proportion of *Paragonimus westermani* infections declined with age, while *Cysticercus* infections presented an increasing trend with age. **(C)** Main routes of transmission among parasite-infected patients. Four major routes of transmission were concluded here (Figure 3C): unclean diets (46.03%, 190/412); traveling abroad (6.78%, 28/412); contacting infected water (2.10%, 9/412); raising animals (1.19%, 5/412); and unknown infective ways (43.93%, 181/412).

These patients were further categorized into nine age groups (Figure 3A and Table 3). The male vs. female ratio was 1.48:1 with the age of 95% of patients ranging from 21 to 70 years. It was worth noting that there was a significant difference in the prevalence of the top six parasitic infections among different age groups (χ^2^ = 64.1, df = 30, *P* < 0.001) (Figure 3B). The incidence of *Paragonimiasis* has declined with age over recent years, but *Cysticercosis* showed an increasing trend with age (Figure 3B). There were four major routes of transmission in the current study (Figure 3C): intake of contaminated food (46.03%, 190/412); traveling to epidemic areas abroad (Africa) (6.78%, 28/412); drinking or contacting parasites within contaminated water (2.10%, 9/412); contact with parasite-infected animals (1.19%, 5/412). Among these infected patients, the major transmission route was contaminated diets/uncooked food, especially uncooked fish (45.45%) and shrimp/crab (21.21%) (Table 4). Importantly, for nearly half of the patients, the routes of transmission could not be determined (43.93%, 181/412).

**Table 3:** Results of laboratory tests of the parasite-infected patients.

	**Total**	**Male**	**Female**	**P value**
Gender	412	246	167	
Age [Median, IQR]	39 (37, 42)	42 (29, 52)	38 (29, 52)	0.5803
Red blood cell count (× 10^12^/L) [Median, IQR]	4.46 (4.39, 4.52)	4.70 (4.41, 4.95)	4.19 (3.88, 4.42)	< 0.0001
White blood cell count [Median, IQR]	7.28 (6.80, 7.60)	7.61 (5.84, 9.59)	6.70 (5.21, 8.50)	0.0036
Neutrophil count (× 10^9^/L) [Median, IQR]	3.39 (3.14, 3.60)	3.50 (2.72, 4.60)	3.10 (2.20, 4.12)	0.0089
Neutrophilic granulocyte percentage (%) [Median, IQR]	49.95 (47.67, 52.23)	49.50 (37.63, 60.95)	50.15 (37.60, 60.60)	0.6709
Lymphocyte count (× 10^9^/L) [Median, IQR]	1.90 (1.80, 1.98)	1.90 (1.49, 2.43)	1.90 (1.60, 2.28)	0.9217
Lymphocyte percentage (%) [Median, IQR]	26.75 (25.10, 28.23)	25.79 (10.12, 51.58)	28.71 (10.46, 46.96)	0.0091
Eosinophil count (× 10^9^/L) [Median, IQR]	0.58 (0.42, 0.70)	0.59 (0.13, 2.32)	0.46 (0.10, 1.59)	0.2095
Eosinophil percentage (%) [Median, IQR]	8.75 (6.50, 11.60)	9.10 (1.90, 27.78)	7.90 (1.78, 24.33)	0.4205
Blood platelet count (× 10^9^/L) [Median, IQR]	209.00 (201.00, 218.29)	203.00 (171.00, 242.00)	214.50 (180.50, 261.50)	0.0332
Hemoglobin concentration (g/L) [Median, IQR]	136.00 (133.00, 137.82)	144.00 (134.00, 152.00)	125.00 (117.00, 133.00)	< 0.0001

Results of blood routine examination of parasite-infected patients were shown. Eosinophil count and eosinophil percentage elevated among male and female patients, but no significant difference was identified between them.

**Table 4:** Patients infected for unclean diets.

	**Number of patients**	**Proportion of patients infected for unclean diet**
Uncooked shrimp and crab	42	21.21%
Uncooked fish	90	45.45%
Spiral shell	4	2.02%
Bullfrog	3	1.52%
Beef	6	3.03%
Uncooked snake flesh	9	4.55%
Other	44	22.22%

Routes of transmission of unclean diets among parasite-infected patients were classified into seven ways. Eating uncooked food, especially eating uncooked fish, became the dominant reason for infection.

### Parasitic species and epidemic trends over the study period

**Figure 4:**
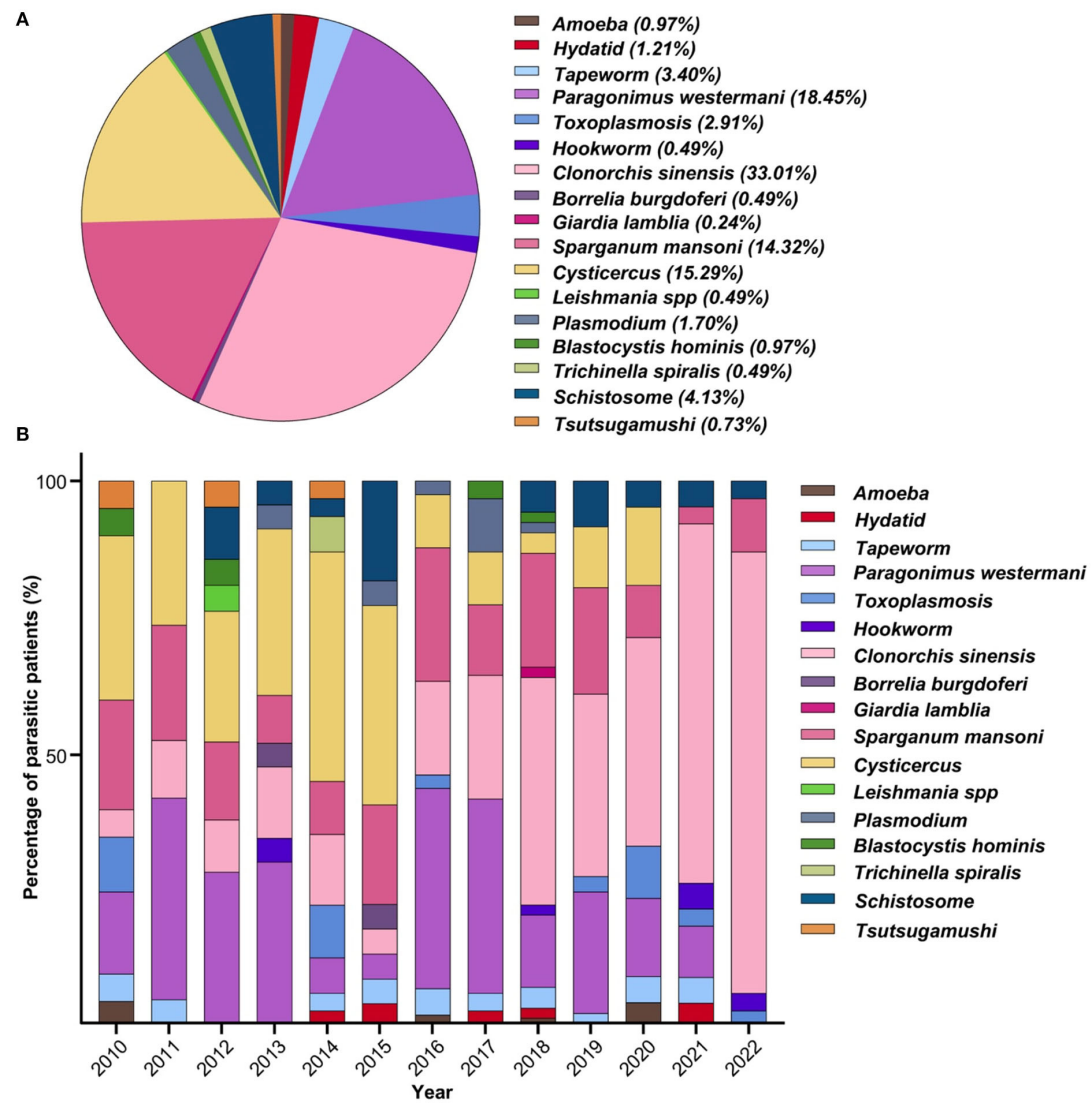
Parasitic species and epidemic trend. **(A)** Constituent ratio of parasitic species. A total of 17 types of parasites were detected in patients. **(B)** Profile of every-year parasitic infections. Food-born parasites (*Clonorchis sinensis, Paragonimus westermani, Cysticercus*, and *Sparganum mansoni*) became the dominant parasitic species with rare infections of soil-transmitted parasites.

A total of 17 types of parasites were detected in these infected patients (Figure 4A). *Clonorchis sinensis* (33.01%, 136/412) was the most common parasite detected, followed by *Paragonimus westermani* (18.45%, 76/412), *Cysticercus* (15.29%, 63/412), *Sparganum mansoni* (14.32%, 59/412), *Schistosome* (4.13%, 17/412), *Tapeworm* (3.40%, 14/412), *Toxoplasmosis* (2.91%, 12/412), *Plasmodium* (1.70%, 7/412), *Hydatid* (1.21%, 5/412), *Amoeba* (0.97%, 4/412), *Blastocystis hominis* (0.97%, 4/412), *Tsutsugamushi* (0.73%, 3/412), *Trichinella spiralis* (0.49%, 2/412), *Borrelia burgdorferi* (0.49%, 2/412), *Hookworm* (0.49%, 2/412), *Leishmania spp* (0.49%, 2/412), and *Giardia lamblia* (0.24%, 1/412). The number of these patients who had multiple parasitic infections with either two, three, or four parasites were 39, 6, or 2, respectively. The total superinfections accounted for 11.4% of the total infections.

The profiles of annual parasitic infections have been analyzed, and the FBPs (*Clonorchis sinensis, Paragonimus westermani, Cysticercus, Sparganum mansoni*) became the dominant parasitic species over the period of the study (Figure 4B). Among all 17 types of parasites, there was a trend toward a higher incidence of *Cysticercus* infection in 2010 (30.00%, 6/20), 2011 (26.32%, 5/19), 2012 (23.81%, 5/21), 2013 (30.43%, 7/23), 2014 (41.94%, 13/31), and 2015 (36.36%, 8/22). Moreover, *Clonorchis sinensis* infection increased during the most recent 6 years of the study, e.g., 2017 (22.58%, 7/31), 2018 (41.51%, 22/53), 2019 (33.33%, 12/36), 2020 (38.10%, 8/21), 2021 (66.67%, 42/63), and 2022 (80.65%, 25/31). Notably, *Paragonimus westermani* infection rates remained relatively stable over the study period, as such: 2011 (36.84%, 7/19), 2012 (28.57%, 6/21), 2013 (30.43%, 7/23), 2016 (36.59%, 15/41), and 2017 (35.48%, 11/31). *Cysticercus* infections were persistently high from 2010 to 2015: six in 2010, five in 2011, five in 2012, seven in 2013, 13 in 2014, and eight in 2015, while other parasitic species were sporadically detected over the period from 2012 to 2020 with less than three patients each year.

### Laboratory testing and evidence of parasitic infection used for diagnosis

**Figure 5:**
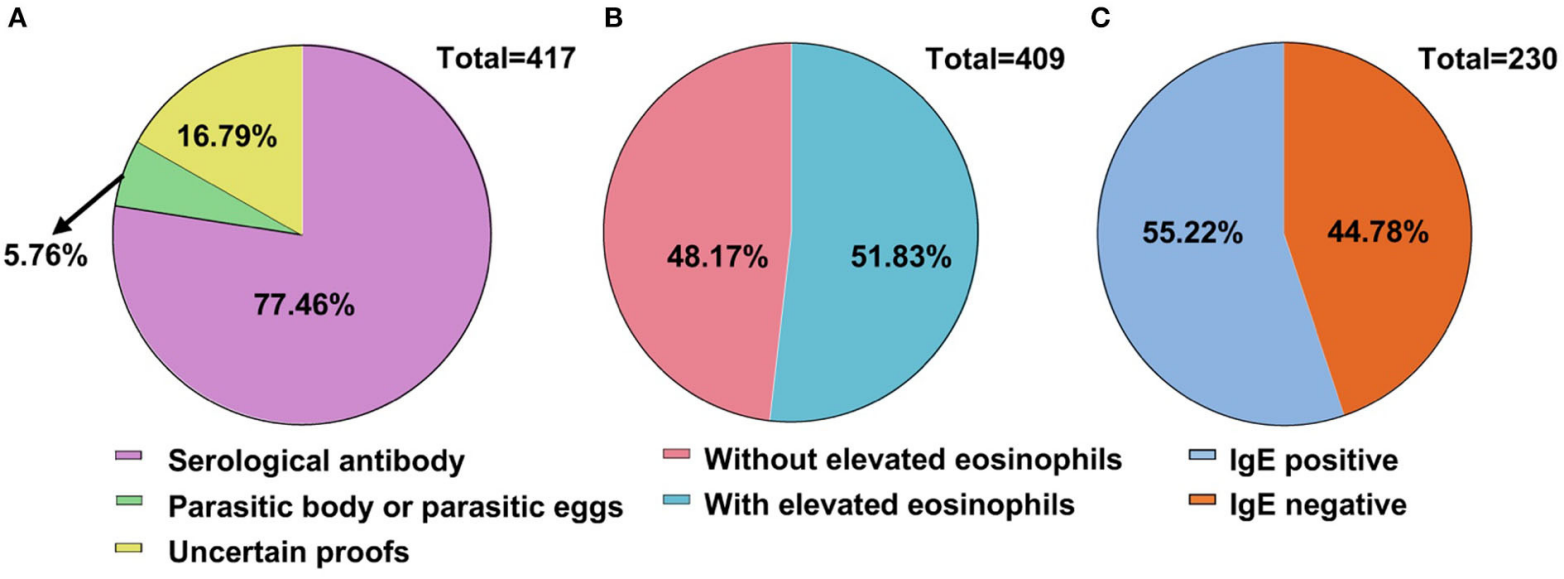
Evidence of parasitic infection for diagnosis. **(A)** Evidence of parasitic infection for diagnosis. Definite evidence of diagnosis had been found in 343 cases, accounting for 83.22%. **(B)** Eosinophilia and parasitic infection. Two hundred and twelve (51.83%) among the total patients were detected with elevated eosinophil count. **(C)** Immunoglobulin E (IgE) and parasitic infections. IgE detection was conducted in 230 patients with more than half (55.22%) showing positive parasitic antibodies (127 cases).

Full blood count data are summarized in Table 3. Eosinophil count (×10^9^/L) and eosinophil percentage (%) were both elevated in male and female patients but without a significant difference (Supplementary Figure 1). There was a significant difference in the eosinophil count, but not eosinophil percentage, among different age groups (*P* < 0.01) (Supplementary Figure 1A) (*P* = 0.05) (Supplementary Figure 1B). Apart from eosinophil counts/percentage, all other hematological parameters were within the normal range for these full blood counts. Definitive evidence of parasitic infection for diagnosis was found in 343 patients (83.22%, 343/412). Serological parasite antibodies within 319 patients (77.61%, 319/412) were detected. Parasitic bodies, such as proglottids, were detected in 18 patients, and eggs of parasites were found in five patients. Sixty-six patients (16.02%) were diagnosed by effective anti-parasite treatments (Figure 5A). Notably, parasite-infected patients frequently exhibit early signs of abnormal blood cytometry, which are prone to be misdiagnosed as hematological diseases. There was a correlation between eosinophil counts and positive antibody titer for 212 parasite-infected patients (Figure 5B). More than half of the patients (127/230, 55.22%) who presented with increased serum IgE had positive parasitic antibody titers (Figure 5C).

### Periods from onset to diagnosis

The periods from the first symptoms to diagnosis among the top four parasites (*Clonorchis sinensis, Paragonimus westermani, Cysticercus*, and *Sparganum mansoni*) are summarized in Table 5. Surprisingly, 38.57%, 25.0%, and 28.3% of patients infected with *Paragonimus westermani, Cysticercus*, or *Sparganum mansoni* were diagnosed after more than 6 months since they had obvious symptoms (Table 5). Moreover, six *Sparganum mansoni-*infected patients had experienced headaches since childhood, but the diagnosis was delayed by >10 years until these patients sought consultations in our hospital. Among these six patients, three were misdiagnosed as epileptic seizures, and the other three patients were misdiagnosed as rheumatoid arthritis. Therefore, in these six patients, the proper effective treatment was substantially delayed for > 10 years post parasitic infections.

**Table 5:** Periods from onset to diagnosis among *Clonorchis sinensis, Paragonimus westermani, Cysticercus* and *Sparganum mansoni*.

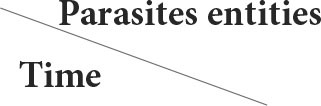	***Clonorchis sinensis***	***Paragonimus westermani***	***Cysticercus***	***Sparganum mansoni***
~2 weeks	33 (40.74%)	9 (12.86%)	26 (43.33%)	15 (28.30%)
~1 month	26 (32.10%)	1 (1.43%)	12 (20.00%)	12 (22.64%)
~6 month	18 (22.22%)	33 (47.14%)	7 (11.67%)	11 (20.75%)
~1 year	1 (1.23%)	7 (10.00%)	6 (10.00%)	4 (7.55%)
>1 year	3 (3.70%)	20 (28.57%)	9 (15.00%)	11 (20.75%)
Total	81	70	60	53

The periods from onset to diagnosis of the most common four parasites (*Clonorchis sinensis, Paragonimus westermani, Cysticercus* and *Sparganum mansoni*) were summarized above.

## Discussion

Parasitic infection is still a public health problem with a huge economic burden, particularly within developing countries. Although great advances have been achieved in fighting parasitic infection over the past 60 years (1), the transmission of parasites remains a challenge worldwide.

The present study demonstrates the clinical characteristics and pathogen spectra of parasitic infections over the latest 13 years from a tertiary teaching hospital in Shanghai. During the most recent 30 years, the incidence of parasitic infection within Shanghai and many other areas of Eastern China has fallen dramatically as a consequence of improved living standards, resulting in lower awareness of parasitic infection within the differential diagnosis, further leading to delayed diagnosis and consequent definitive treatment. Consequently, within most hospitals in Shanghai, the parasitic infection clinical service has been suspended. Though the present study is a single-center investigation, the patients suffering from parasitic infections were not only from Shanghai but also from its surrounding provinces such as Zhejiang, Jiangsu, Anhui, and Jiangxi, accounting for 36.40% of patients, while the patients from other more distant provinces accounted for 28.64%. A previous study in Zhejiang demonstrated that the infection rate of *Clonorchis sinensis* from the consumption of fish was low (8) and the prevalence of soil-derived parasites decreased dramatically from 22.84% (9) in 2004 to 2.12% at the national level (8). An investigation from 2012 to 2015 demonstrated that the infection rate of soil-borne nematode infections was <5% in Hefei City, Anhui province (10). *Clonorchis sinensis* and *Paragonimus westermani* were the two main types of foodborne parasites in Jiangxi province (11). There have been no recent investigations into the evolution of the spectra of parasitic infections within hospitals in Shanghai. Therefore, parasitic infection data from Shanghai Ruijin Hospital, which keeps the parasitic infection clinical service till now, provide important information on the prevention and control decision process concerning parasitic infection in Shanghai. Currently, imported parasitic diseases have increased in recent years as a consequence of more frequent international communications and cooperation between China and other countries in Africa. Food-born parasites (FBPs) are still challenges for successfully controlling the prevalence of parasitic diseases. In our study, the infection rate of the FBP, *Clonorchis sinensis*, was observed to peak in 2021, and eating raw sliced fish was the main source of the infection, likely reflecting changes in popular dietary preferences.

A further explanation for this observation is that intermittent lockdown was implemented in many regions of China following the COVID-19 pandemic (12) from 2020. Citizens were not able to dine out as usual and ordering take-out food became more frequent. The surge in orders might have led to half-cooked food (13). There is no solid evidence to definitively explain the phenomenon that the incidence of *Clonorchis sinensis* infection substantially increased in 2021, but such an observation invites speculation that the source of *Clonorchis sinensis* infection was mainly a consequence of reduced hygiene and inadequately cooked freshwater fish from takeaway sources.

In addition, a trend toward a rise in *Clonorchis sinensis* infection during the most recent 6 years was observed in this study. However, considering that it is difficult to determine the exact time when the patient was initially infected, which may be many years ago, this result can only indicate the increase in the number of infected patients with *Clonorchis sinensis* that have been treated during recent years.

The Chinese government attaches great importance to the prevention and treatment of parasitic diseases. Prior to 2021, China had eliminated *Filariasis* and *Malaria* (14). In addition, other parasitic diseases such as *Schistosomiasis, Hydatid* disease, and geohelminths have been endemic or sporadic (15). *Schistosomiasis* infections have been reduced due to effective prevention and treatment strategies during the most recent 10 years (16, 17). Guangxi and Shanghai, Guangdong, and Fujian provinces have now achieved the national criteria for *Schistosomiasis* elimination (14). However, it is still an endemic disease in Anhui, Jiangxi, Hunan, Sichuan, and Yunnan provinces (1). From 2006 to 2018, the average infection rate of soil-borne parasites decreased from 20.88% to 1.29% (18, 19). Although the infection rate of soil-derived parasites has become very low, they are still threats to health (15). A high infection rate of soil-born parasites still existed in Yunnan (11.83%), Hainan (10.9%), Chongqing (8.68%), Sichuan (6.56%), and Guizhou (4.69%) in 2018 (19). As for foodborne parasites, *Clonorchis sinensis* and other foodborne parasitic diseases also have posed a great threat to food safety in China (19). *Clonorchis sinensis* has shown a high incidence in two major epidemic zones, namely, Heilongjiang, Jilin, Liaoning provinces (Northeast China), and Guangdong, Guangxi provinces (Southwest China). A survey revealed that the infection rate of *Clonorchis sinensis* in a key population of Nanning, Guangxi from 2016 to 2020 was 30.66% (20). Surveys conducted in Guangdong from 2006 to 2012 revealed an increasing trend in the infection rate and intensity of *Clonorchis sinensis* (21, 22). An investigation of *Clonorchis sinensis* carried out in 2016 across 15 provinces in China also demonstrated that 89.37% of the infected people were distributed in Jilin, Heilongjiang, Guangdong, and Guangxi provinces (23). *Tapeworm* is the most harmful foodborne parasite worldwide. Infection with *Tapeworm* can jeopardize organs and tissues and might lead to hemiplegia, epileptic seizure, and heart arrest. In 2015, total infections with *Tapeworm* were about 370,000 and the infection rate was 0.06% in China. Tibet, Sichuan, and Yunnan provinces were the top three regions with high *Tapeworm* infections (24). *Paragonimus westermani* is a type of foodborne parasite that mainly causes pulmonary lesions and the primary endemic areas of *Paragonimiasis* are Heilongjiang, Jilin, Liaoning and Henan, Jiangsu, Zhejiang, and Fujian (25).

A previous study has demonstrated that *Clonorchis sinensis* showed a high incidence in Northeast China (Heilongjiang, Jilin, Liaoning) and Southern China (Guangdong and Guangxi) (25). Our study highlights that *Clonorchis sinensis* has also become the leading cause of parasitic infections in Shanghai during the most recent 3 years, instead of *Paragonimus westermani* (26).

In fact, *Clonorchis sinensis* is not only endemic in China, but also in Korea, Russia, and Vietnam. Investigations conducted in Korea showed that people aged 50–59 were the highest risk population for *Clonorchis sinensis* infection (27), while people aged 20–40 were the most vulnerable population in our study.

Eosinophilia is used as a sign of parasitic infection necessitating further confirmation of parasitic infection (28). The present study showed that 51.83% (212/409) of patients were found to have elevated eosinophils, while a study conducted in India only found 10.6% (66 cases) with relative eosinophilia among 621 parasitic infections (29).

With the rapid improvement in the economy and transportation, geographical boundaries of foodborne parasites have been broken. Though there are a substantial number of individuals infected with parasites, most healthcare providers are currently not familiar with parasitic diseases (30), which consequently leads to the neglect of consideration of parasitic diseases and delay in diagnosis. Diagnosis of parasitic infection should not only depend on parasitic antibody detection but also needs to apply new diagnostic methods such as molecular biological methods and next-generation sequencing (NGS) (30). At present, the effectiveness of anti-parasitic drugs is limited clinically, as well as the evaluation system of therapeutic effect. Praziquantel has been used as a key medication for treating parasitic infections for about 50 years. However, the growing number of praziquantel-resistant parasites is an increasing threat in this field. The resistance of *Schistosoma* to praziquantel in epidemic areas has drawn much attention recently (31). Monitoring drug resistance in high transmission areas, where treatment failure often happens, should be initiated (32). New therapies are needed to confront the urgent threats to drug-resistant parasites and emerging parasites (33). The information from our current study may offer the following points for the control and prevention of parasitic infection in future.

Parasitic infection cannot and should not be ignored by clinicians, particularly specialists in infectious diseases. Our current study demonstrates that in many patients, parasitic infections had not been infected recently, providing challenges in the real world.

First, the lack of specific diagnostic kits for parasites in some areas may be the dominant reason, as the diagnostic kits for parasites are only available centrally at the CDC. Second, the diagnosis and treatment services for parasitic diseases have either not been established or have been closed in many hospitals as a consequence of the declining prevalence of parasitic infection. Moreover, most food-born parasitic diseases with non-specific clinical symptoms are easily confused with other medical diseases, and the epidemiological history collection is incomplete or lacks specificity, enhancing the difficulty of diagnosis (34).

The current study provides an alarm for clinicians to be prepared for potential parasitic infection(s), although the incidence is rather low in the real world for advanced and well-developed regions and/or countries. In addition, parasitic infections should be considered for any patients with fever of unknown origin, raised IgE, and eosinophilia. Currently available specific diagnostic kits for parasites in China can only identify nine types of serum antibodies of parasites: *Schistosome, Clonorchis sinensis, Toxoplasmosis, Paragonimus westermani, Blastocystis hominis, Sparganum mansoni, Tubular nematode, Fasciola*, and *Hydatid*. More advanced and versatile technology should be developed for easy and quick diagnosis of parasitic infections, which could offer accurate and early diagnosis and subsequent effective treatments for these parasite-infected patients, particularly for certain regions with a high risk of parasitic infections. Additionally, setting up specialized disease outpatient departments for parasitic diseases in some regions is necessary and urgent. Diagnosis of parasitic infection should also consider different age groups with different susceptibilities.

Importantly, a system of therapeutic effect evaluation and specific indicators for the outcome should be developed. Praziquantel, as a broad-spectrum anti-parasitic agent specific for parasitic infection, has been widely applied for nearly 40 years (35). However, the medical history of many of the patients in our study lacked prognostic biomarkers, preventing us from accurately evaluating the outcome of anti-parasitic treatment. Consequently, as there were no specific prognostic markers for parasitic diseases, it was difficult to distinguish if those parasite-infected patients were freshly infected or not. Thus, new biomarkers for accurately monitoring treatment outcomes are desirable for our future effective management.

Due to the occult features of many parasitic infections, definite evidence of parasitic infections in a small number of parasite-infected patients was difficult to find. Given this issue, sensitive biomarkers specific to parasitic infections are worthy to be investigated and it is urgent to develop novel parasite kits for early, rapid, and accurate diagnosis to cope with the current situation with constantly emerging parasites.

The limitation of our study is that this survey is just a single-center study within Shanghai. A multicenter investigation should be implemented within the different regions of China, especially within high-risk zones in future.

## Conclusion

Compared to soil-born parasites, food-born parasites have become more prevalent in recent years in Shanghai. Our current study demonstrates the transitional change in the prevalence of parasitic infection over the latest 13 years in a single center in Eastern China. The incidence of parasitic infections peaked in 2021 over the 13-year period, and the dominant parasitic species switched from a soil origin to foodborne. It is worth noting that *Clonorchis sinensis* has become the main species diagnosed in recent years. Although great achievements have been accomplished in the past few decades, the parasitic infection should not be ignored by clinicians to minimize misdiagnosis, which offers the best option for these patients. Although eosinophilia and raised IgE, when present, are good indicators for screening parasitic infection, they are not sensitive enough for diagnosing parasitic diseases. More advanced and versatile technologies should be developed for easy and quick diagnosis of parasitic infections, which could offer accurate diagnosis and subsequent effective and timely treatment for these parasite-infected patients, particularly for certain regions with a high risk of parasitic infections. Additionally, in the course of treatment and post-treatment, a system for the assessment of therapeutic effects and specific biomarkers for evaluating prognosis should be developed for the patients to promote precise management.

## Data availability statement

The original contributions presented in the study are included in the article/Supplementary material, further inquiries can be directed to the corresponding author/s.

## Ethics statement

The studies involving human participants were reviewed and approved by Ethics Committee of Ruijin Hospital, Shanghai Jiao Tong University School of Medicine. Written informed consent to participate in this study was provided by the participants' legal guardian/next of kin. Written informed consent was obtained from the individual(s), and minor(s)' legal guardian/next of kin, for the publication of any potentially identifiable images or data included in this article.

## Author contributions

Conceptualization: XX, QX, and DShi. Data curation and methodology: JZ, JX, and WT. Roles/writing - original draft: JZ and XX. Writing - review & editing: RM, DSha, JL, ZL, and XW. All authors have access to the study data and have reviewed and approved the final manuscript.

## Funding

This study was supported by the National Natural Science Foundation of China (Nos. 82170619, 81970544, 82070604, 81770587, 81770578, and 81900527), the 3-Year Public Health Action Plan (2020–2022) of Shanghai (No. GWV-10.1-XK13), the Shanghai Municipal Key Clinical Specialty (shslczdzk01103), the Shanghai Ruijin Hospital Clinical Skills and Innovations (2018CR005), the Shanghai talent development fund (2020097), the Shanghai Rising Stars of Medical Talent Youth Development Program Outstanding Youth Medical Talents (SHWJRS(2021)-99), the Shanghai Outstanding Academic Leader Youth Program (20XD1422600), the Shanghai Sailing Program (No. 19YF1429200), and Shanghai Pujiang Talent Program (21PJD041).

## Conflict of interest

The authors declare that the research was conducted in the absence of any commercial or financial relationships that could be construed as a potential conflict of interest.

## Publisher's note

All claims expressed in this article are solely those of the authors and do not necessarily represent those of their affiliated organizations, or those of the publisher, the editors and the reviewers. Any product that may be evaluated in this article, or claim that may be made by its manufacturer, is not guaranteed or endorsed by the publisher.
