# Supplementary material for: Clinical characteristics and pathogen spectra of parasitic infections in a tertiary hospital of Shanghai: A 13-year retrospective study
Source: Front Public Health. 2022 Sep 28;10:993377. doi: 10.3389/fpubh.2022.993377 (PMC9554607; doi:10.3389/fpubh.2022.993377)
Supplement: Supplementary file 2 [file Image_1.pdf]

Supplementary materials

Supporting figure

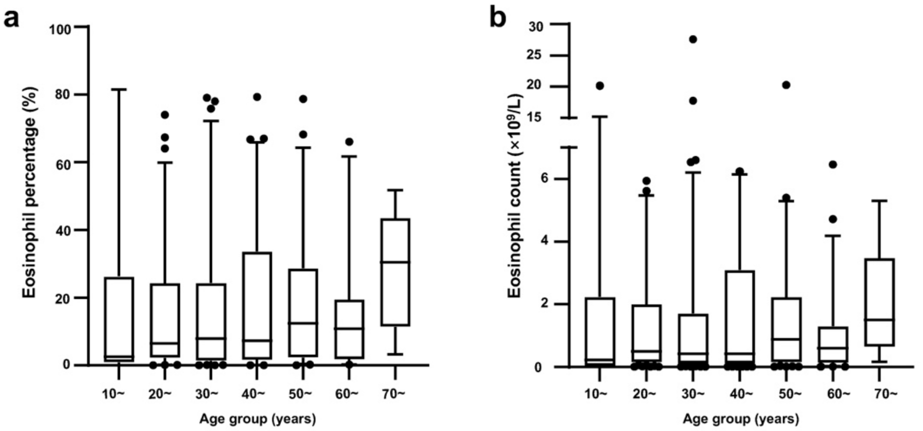

Supporting figure S1. Eosinophil percentage (%) and eosinophil count (×10^9/L) among different age groups. Eosinophil count (×10^9/L) and eosinophil percentage (%) both elevated in male and female patients but no significant difference was displayed (**Figure S1**). There was a significant difference in the eosinophil count among different age groups (P<0.01) (**Figure S1a**), but no significant difference was found in eosinophil percentage (P=0.0506) (**Figure S1b**).
